# Forming-Free Tunable Analog Switching in WO_x_/TaO_x_ Heterojunction for Emulating Electronic Synapses

**DOI:** 10.3390/ma15248858

**Published:** 2022-12-12

**Authors:** Chandreswar Mahata, Juyeong Pyo, Beomki Jeon, Muhammad Ismail, Myounggon Kang, Sungjun Kim

**Affiliations:** 1 Division of Electronics and Electrical Engineering, Dongguk University, Seoul 04620, Republic of Korea; 2 Department of Electronics Engineering, Korea National University of Transportation, Chungju-si 27469, Republic of Korea

**Keywords:** WO_x_/TaO_x_ bilayer, gradual resistive switching, synaptic plasticity, short-term plasticity, long-term potentiation, spike-rate-dependent plasticity

## Abstract

In this work, the sputtered deposited WO_x_/TaO_x_ switching layer has been studied for resistive random-access memory (RRAM) devices. Gradual SET and RESET behaviors with reliable device-to-device variability were obtained with DC voltage sweep cycling without an electroforming process. The memristor shows uniform switching characteristics, low switching voltages, and a high R_ON_/R_OFF_ ratio (~10^2^). The transition from short-term plasticity (STP) to long-term potentiation (LTP) can be observed by increasing the pulse amplitude and number. Spike-rate-dependent plasticity (SRDP) and paired-pulse facilitation (PPF) learning processes were successfully emulated by sequential pulse trains. By reducing the pulse interval, the synaptic weight change increases due to the residual oxygen vacancy near the conductive filaments (CFs). This work explores mimicking the biological synaptic behavior and further development for next-generation neuromorphic applications.

## 1. Introduction

To improve beyond the conventional memory storage device for high-density applications, resistive random-access memory (RRAM) consisting of memory cells with a simple metal-insulator-metal structure has attracted considerable attention [1,2,3]. Several researchers have extensively studied the resistive switching effects of next-generation non-volatile memory applications due to their low power consumption, high operating speed, and compatible fabrication steps [2,4,5]. RRAM structure consists of a switching layer sandwich between two electrodes, and a transition from a high-resistive state (HRS) to a low-resistive state (LRS) due to the oxygen vacancy (V_O_), and the migrations and recombination of oxygen ions (O^−^) [6]. The rupture and formation of the conductive filaments (CFs) that form during electroforming are controlled mainly by the oxygen vacancy creation in the switching materials [7,8]. Gradual resistance changes in the memristor with a high ON/OFF ratio can be controlled under DC and pulse conditions for the applications of analog neuromorphic devices [9,10,11]. Therefore, the motivation for implementing the synaptic function in resistive switching devices is to mimic artificial synapses by a graded oxygen content switching layer. Compared to other transition metal oxides such as Al_2_O_3_, TiO_2_, HfO_2_, and ZrO_2_, the oxygen vacancy formation is well controlled in WO_x_ and TaO_x_ [12,13,14,15,16,17,18,19].

Kim et al. have described uniform switching ability in the Pt/WO_x_/W memristor with multi-bit memory applications by the movement of controlled oxygen vacancies due to the intrinsic property of WO_x_ [20]. Moreover, it was earlier reported that the rectification of the resistive switching performance was achieved in Pt/WO_x_/Au RRAM due to the work function asymmetry. Electric-field-induced oxygen vacancy electromigration was found to be responsible for SET and RESET behavior [21].

It has been reported that TaO_x_ can generate oxygen vacancies easily, which is beneficial for forming strong conductive filaments for non-volatile memory applications [16,22]. However, an additional oxygen exchange layer can help gradual switching due to the redox reaction under an external electric field [17]. Modified graded oxygen content in the TaO_x_ switching layer has demonstrated that the conical shape of CFs can control the formation and rupture in an effective way for high-performance RRAM devices [18]. Ma et al. predicted that in TiN/TaO_2.0_/TiN RRAM structures, the electroformation of CFs is less dominated by the oxygen ions reacted on the electrode [19]. Sugawara et al. have described a TaO_x_ bilayer memristor with different concentrations of oxygen, where the movement of oxygen ions define the resistance transition, which leads to analog resistive switching [6]. Park et al. also described a TaO_2−x_/Ta_2_O_5−x_-based resistive switching device where the SET process is controlled by the V_O_ migration from the bottom TaO_2−x_ layer [8].

Bilayer Pt/HfO_2_/TaOx/Pt memristor devices show hysteresis behavior, and multiple resistance states are achieved by controlling the conductive filaments at the HfO_2_/TaO_x_ interface [2]. In the Ta/WOx/TaOx/Pt bilayer, WO_x_ was used as a redox layer, and the WO_x_/TaO_x_ interface controls the movement of oxygen vacancies in the TaO_x_ layer that results in gradual resistive switching [17]. Overgrowth CFs were also reduced due to the non-existence of redox reactions (because of the presence of the Al_2_O_3_ layer in the W/Al_2_O_3_/TaO_x_/TiN memory structure).

In this study, the gradual conductance change was controlled in the sputtered deposited WO_x_ (50 nm)/TaO_x_ (5 nm) bilayer thin film. Therefore, a detailed understanding of the memory performance is associated with the multilevel resistance and modulation of conductance under DC and pulse bias conditions. Critical synaptic functions were mimicked with stable multistate memory characterizations. The WO_x_ redox reactive layer was inserted between the ITO electrode and TaO_x_ switching layer. Thus, the biological synaptic function has been demonstrated, including the spike-rate-dependent plasticity (SRDP), short-term plasticity (STP), long-term plasticity (LTP), and paired-pulse facilitation (PPF). The improved synaptic properties of the ITO/WO_x_/TaO_x_/TiN memristor device makes it a promising candidate for neuromorphic computing.

## 2. Materials and Methods

The memristors were fabricated with titanium nitride (TiN) as the bottom electrode, deposited by DC sputter using a Ti (99.99%) target. The working pressure was kept at 1 mTorr (Ar, 19 sccm, and N_2_, 1 sccm) with 350 W power. First, an insulating layer of ~5 nm TaO_x_ was formed using DC sputter at 5 mTorr working pressure under a gas mixture of Ar, 20 sccm, and O_2_, 6 sccm. The second layer of WO_x_ (~50 nm) was deposited on TaO_x_ by RF sputter (at 80 W) from a W target using a gas mixture of Ar, 20 sccm, and O_2_, 6 sccm. Using photolithography, square patterns of 100 µm × 100 µm were made. Finally, indium tin oxide (ITO) top electrodes were achieved by the lift-off process. Using RF sputter at 60 W, ITO was deposited from a commercial ITO target (99.99% purity), under the gas pressure of 3 mTorr, using Ar. The electrical characteristics were obtained by connecting the bottom electrode to the ground, and external electric fields were applied to the top electrode. The Keithley 4200 SCS semiconductor parameter analyzer and 4225-PMU pulse module were used for DC I-V cycling for multistate memory characterization and to observe synaptic behavior of the ITO/WO_x_/TaO_x_/TiN memristor devices.

## 3. Results and Discussion

### 3.1. Gradual Bipolar Resistive Switching of the ITO/WO_x_/TaO_x_/TiN Device

**Figure 1:**
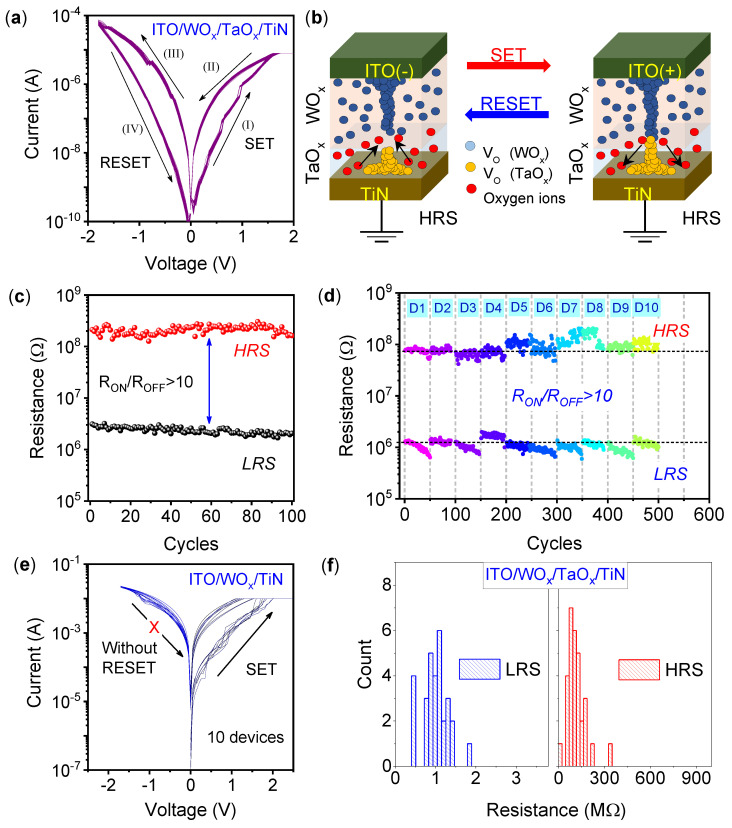
(**a**) Bipolar resistive switching characteristics of the ITO/WO_x_/TaO_x_/TiN device. (**b**) Schematic description of the SET and RESET process showing the formation and annihilation of conductive filament. (**c**) DC endurance characteristics with 100 stable cycles. (**d**) Multiple devices show promising device-to-device reliability with a uniform R_ON_/R_OFF_ ratio. (**e**) I–V characteristics of the ITO/WO_x_/TiN device at the current compliance of 10 mA without RESET characteristics. (**f**) The distribution histogram of HRS and LRS for 30 ITO/WO_x_/TaO_x_/TiN memristors.

Under a positive bias voltage with the current compliance (I_CC_) of 10 µA, the SET process of the ITO/WO_x_/TaO_x_/TiN memristor was observed, as shown in Figure 1a. In step-I, the current increases gradually and reaches the I_CC_. The device is then in a low-resistance state (LRS) in step-II under the sweepback voltage. The device shows forming-free bipolar switching, which is typical intrinsic TaO_x_ behavior for the presence of abundant oxygen vacancies [23]. Generating a large amount of VOs during applications of external electric fields helps the forming-free SET process, which is advantageous for RRAM applications [24]. As electroforming is a destructive process due to chemical reactions, it can change the switching layer’s intrinsic properties and uniformity for device-to-device performance [25]. At negative bias, the RESET process occurs, as shown in step-III and step-IV. The overall gradual transition of the SET and RESET operation in the device predicts the gradual change of conductance, which is promising for multilevel memory storage. Forming-free resistive switching may be attributed to uniformly distributed oxygen vacancies that accumulate and form the CFs under external positive bias [26]. The switching mechanism of the SET and RESET process is described in Figure 1b. In this case, the top WO_x_ layer is believed to act as an oxygen ion reservoir, which further helps the redox reaction at the WO_x_/TaO_x_ interface. The CFs are preferably ruptured near that point, and the gradual switching may be due to trap-assisted-tunneling (TAT) conduction [27]. Therefore, at positive bias, the conduction path is created in the TaO_x_ layer, which consists of oxygen vacancies [2]. In the RESET process, the recombination of oxygen ions that have drifted from the WO_x_ and TaO_x_ layers leads to the rupture of CFs at the WO_x_/TaO_x_ interface [28]. Thus, unlike other transition metal oxides such as Al_2_O_3,_ in which redox reactions are absent, WO_x_ benefits the gradual SET process [29]. Forming-free resistive switching in the amorphous TaO_x_/WO_x_ layer is described by Prakash et al., where the switching mechanism is dominated by the CFs formation and rupture [30]. Furthermore, Wang et al. have described a large number of oxygen vacancies contained in sputtered TaO_x_ [31]. The low resistive TaO_x_ layer shows the bipolar resistive switching characteristics without any forming process. Cyclic DC endurance characteristics up to 100 cycles show a stable memory window, as shown in Figure 1c. Endurance properties for 10 ITO/WO_x_/TaO_x_/TiN memristor devices are shown in Figure 1d. A device-to-device reliability with a R_ON_/R_OFF_ ratio >10 predicts that the memristor is suitable for high-density and gradual resistive switch synaptic devices. Figure 1e shows the I-V characteristics of ITO/WO_x_/TiN devices. As mentioned above, the leakage current was found to be high due to a significant concentration of intrinsic oxygen vacancies in the WO_x_ layer. The ITO/WO_x_/TiN device was set at a very high current compliance of 10 mA. However, due to the formation of very thick CFs, the RESET process cannot be achieved. Multiple ITO/WO_x_/TiN devices were measured to confirm the phenomenon, as shown in Figure 1e. A histogram of LRS and HRS distribution from the resistive switching characteristics of the ITO/WO_x_/TaO_x_/TiN memristor has been plotted in Figure 1e. The average LRS and HRS at the read voltage of −0.2 V are 1.05 MΩ and 110 MΩ, respectively. The well-uniform distribution of 30 memristors shows the device-to-device reliability of the present device.

**Figure 2:**
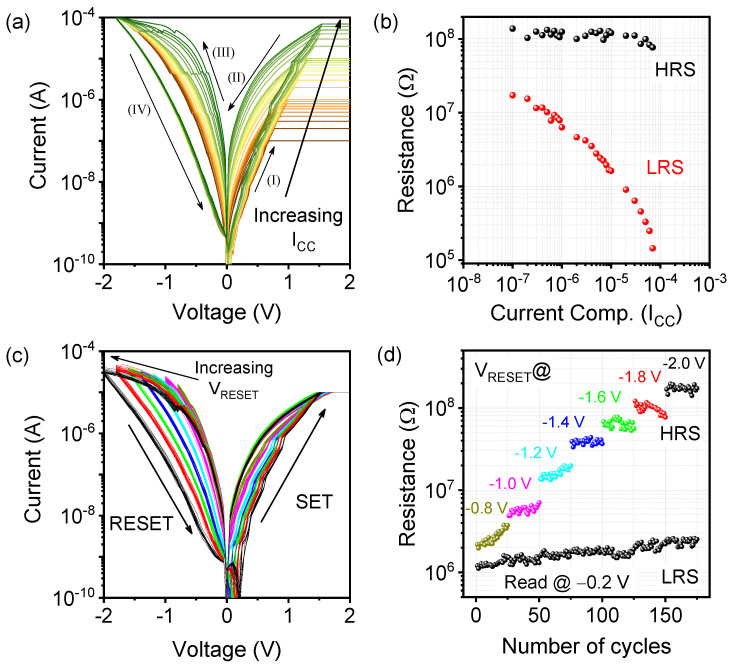
(**a**) Increasing LRS behavior of the ITO/WO_x_/TaO_x_/TiN memristor device by varying the SET current compliance from 1 × 10^−7^ A to 7 × 10^−5^ A. (**b**) Variations of multiple LRSs at each current compliance from 17.2 MΩ to 0.15 MΩ. (**c**) Multilevel memory state achieved by increasing V_RESET_ from −0.8 V to −2.0 V at the current compliance of 1 × 10^−5^ A. (**d**) Endurance characteristics of 7 HRSs under different V_RESET_ values where the I_ON_/I_OFF_ ratio increases gradually from 2.2 to 73.8.

A desirable high-density memory for synaptic devices is achieved by controlling the I_CC_ value during SET operation and the RESET stop voltage, which can modulate the CFs’ size and diameter [32]. Multiple resistance states were obtained by modulating I_CC_ from 1 × 10^−7^ A to 7 × 10^−5^ A during the SET process in the ITO/WO_x_/TaO_x_/TiN memristor, as shown in Figure 2a. The forming and rupture of oxygen vacancies near the CFs at the WO_x_/TaO_x_ interface is controlled by the O^−^ ions’ migrations through redox reactions, as described by Prakash et al. [33]. The low resistance state (LRS) varied from 17.2 MΩ to 0.15 MΩ, as shown in Figure 2b. The gradual increase of the CFs’ diameter caused by increasing I_CC_ was near the WO_x_/TaO_x_ interface, initiating multiple LRSs. Therefore, the ITO/WO_x_/TaO_x_/TiN memristor is suitable for the application of multistate memory devices.

A gradual RESET process was observed when the memristor was subjected to an increasing negative voltage from −0.8 V to −2.0 V at the SET I_CC_ value of 10 µA, as shown in Figure 2c. The ratio of HRS to LRS (I_ON_/I_OFF_) induced by increasing V_RESET_ was increased from 2.2 to 73.8. The gradual recombination of oxygen ions near the CFs controls the thinning of CFs inside the TaO_x_ layer [34]. Linearly increasing the V_RESET_, a uniform change in the HRS, indicates a stable distribution of oxygen vacancies inside the switching layer, which is essential for gradual resistive switching in synaptic devices. The endurance characteristics of each HRS were confirmed by 25 switching cycles read at −0.2 V. The migration of oxygen ions from the WO_x_ layer helps gradual recombination at the WO_x_/TaO_x_ interface.

### 3.2. Synaptic Characteristics under an Identical Pulse Sequence

**Figure 3:**
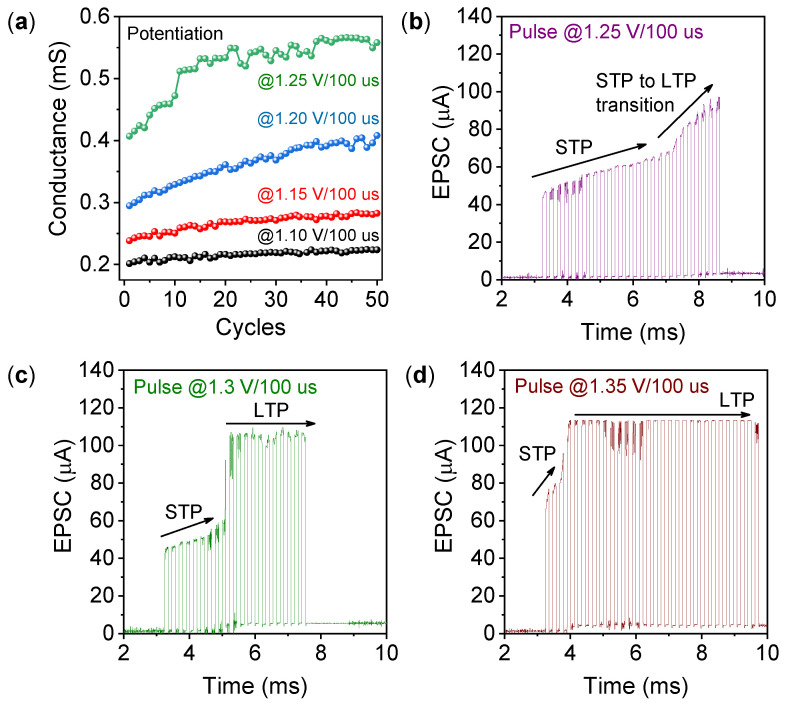
(**a**) Potentiation behavior under an identical pulse sequence of amplitude 1.1 V/100 µs to 1.25 V/100 µs. (**b**–**d**) EPSC obtained in the TO/WO_x_/TaO_x_/TiN memristor during the transition from STP to LTP was observed by increasing the pulse amplitude and number from 1.1 V/100 µs to 1.25 V/100 µs.

Long-term plasticity (LTP) and short-term plasticity (STP) are the critical features of synaptic function [35]. The spike-amplitude-dependent plasticity (SADP) behavior shown in Figure 3a demonstrates that the pulse amplitude highly influences synaptic weight change. Similar characteristics were reported by Hao et al. [36]. Figure 3b–d depict the transition from STP to LTP, depending on the pulse amplitude and spike numbers. As shown in Figure 3b, by applying a pulse amplitude of 1.25 V/100 µs, a slow transition from STP to LTP can be obtained. Whereas, at higher pulse amplitude, a sharp transition occurs, as shown in Figure 3c,d. The EPSC obtained by higher voltage stimuli showed a permanent potentiation behavior with a saturated value up to the applied I_CC_. The currents at the base voltage (+0.2 V) also confirm the phenomenon. This experiment demonstrated that the STP–LTP transition occurred due to repeated pulse stimulations [37]. The EPSC obtained under the repeated pulse sequence and at different amplitudes confirms that the transition occurred after sufficiently thicker CFs were formed. STP behavior is due to the rupture of weak conductive filaments near the WO_x_/TaO_x_ interface. A small number of pre-synaptic pulses produce unstable thin filaments that dissolve quickly after the withdrawal of pulse trains, and the EPSC recovers its original value at the base (read) voltage, as shown in Figure 3b–d. On the other hand, a significant number of oxygen vacancies are created (strong CFs are formed) after increasing the number of stimuli, leading to LTP behavior [38]. The overall EPSC increment of the memristor at LTP is due to the repeated rehearsal of the stimuli, analogous to the biological memory functions. This transition behavior was reported by Chang et al. previously, and is crucial for neuromorphic systems applications [39].

### 3.3. Pulse Frequency-Dependent Synaptic Properties

**Figure 4:**
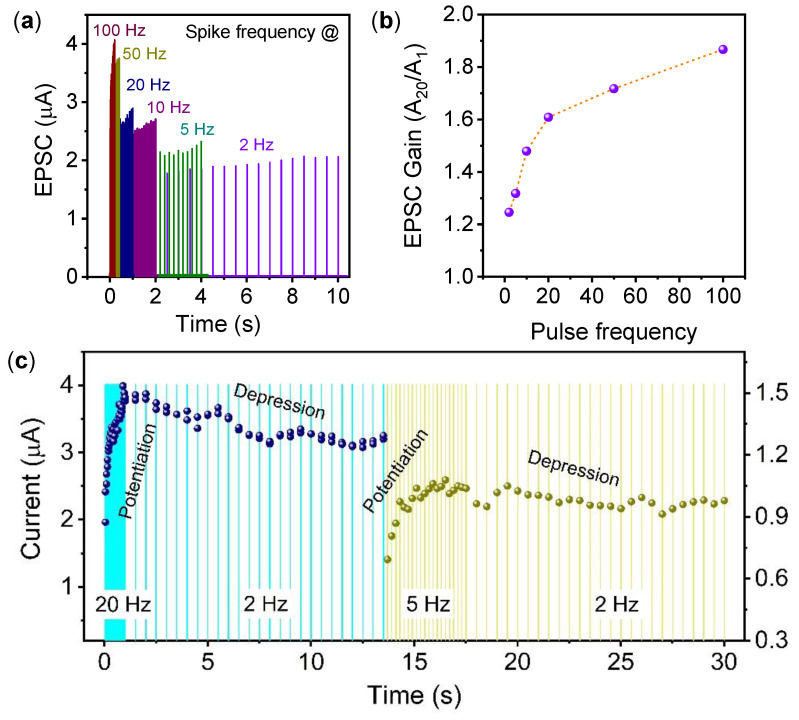
(**a**) Pulse-frequency-dependent current response of the ITO/WO_x_/TaO_x_/TiN memristor with trains of 20 pulses at a frequency range of 100 Hz to 2 Hz with an amplitude of +1.5 V/200 µs. (**b**) EPSC gain was obtained as A_20_/A_1_ by the pre-synaptic pulse sequence at different frequencies. (**c**) Post-synaptic current response at the same voltage amplitude of +1.5 V/200 µs with decreasing frequency.

In neuroscience, tunable synaptic weight change, which depends on the pulse sequence frequency as the spike-rate-dependent plasticity (SRDP), is a necessary learning process [40]. The modulation of EPSC in the ITO/WO_x_/TaO_x_/TiN memristor was monitored by varying the pulse interval, as shown in Figure 4a. The current response was monitored under pulse train frequencies from 2 Hz to 100 Hz with a fixed amplitude and width of +1.5 V/200 µs. Each frequency response was measured after resetting the memristor in DC mode to bring the device into the initial HRS. Under all pulse frequencies, the current increment indicates that the frequencies are less than the V_O_ and O^−^ recombination time [41]. The higher current was achieved with a continuous application of a pulse train with shorter intervals (higher pulse frequency). The increased current response at the higher spiking rate is due to the residual oxygen vacancies near the CFs created by the previous spike influence [40,42]. The short-term plasticity at different frequencies for the ITO/WO_x_/TaO_x_/TiN memristor can act as dynamic filters [42,43,44]. The creation of high-density oxygen vacancies at the WO_x_/TaO_x_ interface with a higher pulse frequency act as the high-pass filtering characteristic. This is similar to parallel fiber synapses in a biological system, as the pre-synaptic pulse train can control the post-synaptic output characteristics [42,45]. As shown in Figure 4a, the memristor can act as a high-pass dynamic filter because a higher EPSC gain is obtained under shorter pulse intervals. The EPSC gain at each frequency was calculated and plotted in Figure 4b. It was calculated from the ratio of A_20_/A_1_, where A_20_ and A_1_ are the peak amplitude of currents after the 20th and 1st pulse, respectively. The EPSC gain of 1.24 at 2 Hz was increased to 1.86 at 100 Hz, indicating that the ITO/WO_x_/TaO_x_/TiN memristor exhibits frequency-dependent synaptic weight modulation. Experience-dependent short-term plasticity was observed, where the post-synaptic current increases and decreases depending on the immediate applications of higher- and lower-frequency pulse trains. The experimental results show that the change in synaptic weight depends on the interval of the spikes. Similar to biological memory, the post-synaptic currents increase at the higher frequency of spike trains. Whereas, at the immediate application of a lower input spike frequency, a synaptic depression was observed without the application of an opposite-polarity pulse sequence [46]. As shown in Figure 4c, a potentiation behavior was observed at a 20 Hz pulse train frequency. Afterward, at a 2 Hz pulse frequency, the currents decrease (depression behavior) at the same pulse amplitude at +1.5 V/200 µs. Similarly, depression behavior was observed at a 2 Hz pulse frequency immediately after a 5 Hz pulse frequency. Unlike the result presented in Figure 4a, the EPSC increases at a 2 Hz pulse frequency due to the application of an individual set of pulse trains after bringing the memristor to its RESET (HRS) state. Therefore, this phenomenon mimics the bio-realistic function where the synaptic weight changes depend on the previous pulse stimulation frequency. The migrated oxygen ions from the WO_x_ layer in the structure also trigger the recombination of oxygen vacancies and oxygen ions. Thus, the ITO/WO_x_/TaO_x_/TiN memristor can mimic the history-dependent plasticity where synaptic weight change depends on the previous experience. This behavior is similar to the paired-pulse depression phenomenon and was also observed in earlier studies [17,36,47].

### 3.4. Paired-Pulse Facilitation (PPF) Emulation

**Figure 5:**
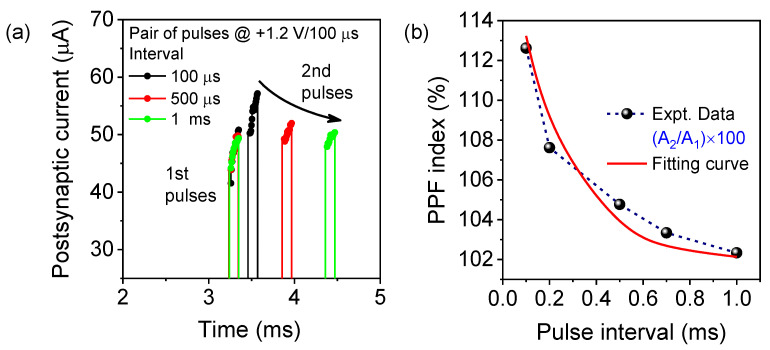
(**a**) Post-synaptic current response observed by applying a paired pulse with the different intervals of 0.1 ms to 1 ms on the ITO/WO_x_/TaO_x_/TiN memristor device. Pre-synaptic pulses were applied with an amplitude of +1.2 V/100 µs. (**b**) Experimental PPF ratios were calculated as a function of the pulse interval plotted as (A_2_/A_1_) × 100% with a simulated curve using the model in Equation (1).

In the short-term plasticity phenomenon, PPF, two consecutive pulse stimuli are applied, and the EPSCs are monitored due to the change in post-synaptic weight. The application of a pre-synaptic paired pulse increased the EPSC amplitude, which was triggered by the second pulse that was higher than the previous one [41,47,48]. As shown in Figure 5a, consecutive paired pulses were applied to the ITO/WO_x_/TaO_x_/TiN memristor with different pulse intervals (Δt) ranging from 0.1 ms to 1 ms with a pulse amplitude of +1.2 V/100 µs. It was found that with increasing the pulse interval, EPSC responses decrease gradually. At the first pulse, the oxygen vacancies are accumulated near the CFs. However, due to the shorter spike interval of 0.1 ms, the oxygen vacancies do not have enough time to recombine with oxygen ions immediately after removing the first pulse. Therefore, the remaining oxygen vacancies that accumulated near the CFs and produced a higher EPSC appeared at the second applied pulse, which induced the PPF. Although at a higher spike interval time, the reserved oxygen vacancies have a longer time to recombine with O^−^, resulting in a reduced EPSC amplitude, as shown in Figure 5a. Thus, at the larger time interval, the enhancement of the post-synaptic current at the second pulse is likely to fade away, which is similar to the biological synapse. The PPF index was calculated from (A_2_/A_1_) × 100, which reached 112.6% at a Δt of 0.1 ms, and decays gradually with the increasing pulse interval, as shown in Figure 5b. The experimental data have been fitted with the decay factor to determine the relaxation time constant using Equation (1) [41]:

PPF = C_1_ exp(−Δt/τ_1_) + C_1_ exp(−Δt/τ_2_), (1)

where τ_1_ and τ_2_ are the characteristic relaxation times, Δt is the time interval between two successive pre-synaptic spikes, and C_1_ and C_1_ are the initial facilitation magnitudes. The relaxation time constants τ_1_ and τ_2_ estimated from the simulations were 48 µs and 255 µs, respectively. Therefore, the synaptic weight change due to the short-term plasticity was successfully emulated in the bilayer of the WO_x_/TaO_x_ artificial synapse, confirming potential applications in neuromorphic computing. In Table 1, a detailed comparison of DC electrical parameters is presented, showing that the ITO/WO_x_/TaO_x_/TiN memristor has improved gradual SET and RESET characteristics with reliable device-to-device variability and memory window. Also, the sputtered deposited WO_x_/TaO_x_ switching layer has promising synaptic properties suitable for neuromorphic applications.

## 4. Conclusions

In conclusion, fabricated memristors demonstrated gradual SET/RESET characteristics due to the presence of the WO_x_ oxygen ion reservoir, which promotes the redox reaction at the WO_x_/TaO_x_ interface. The multilevel memory characteristics were observed by varying the SET current compliance and V_RESET_ with a switching ratio >100 under multiple cycling processes. Synaptic behavior was obtained with potentiation characteristics under different pulse amplitudes. Moreover, oxygen migration and redox reactions under pulse application control reliable SRDP and SADP. Therefore, the bilayer-WO_x_/TaO_x--_based memristor is promising for the use in high-density memory integration in low-power synaptic devices for neuromorphic computing.

## Figures and Tables

**Table 1:** Comparison of resistive switching and synaptic properties of the TaO_x_ and TaO_x_-bilayer-based memristor.

Device Structure	*V*_SET_ (V)	*V*_RESET_ (V)	ON/OFF Ratio	Switching Characteristics	Device-to- Device Reliability	Synaptic Properties	Ref.
Ta/TaO_x_/Pt	+0.65 V	–0.6 V	>10	Abrupt	N/A	N/A	[49]
Ir/TaO_x_/W	+2.5 V	–2.5 V	>10	Gradual	N/A	N/A	[30]
W/Ta/TaO_x_/ Pt	+0.75 V	–1.5 V	>10	Gradual	N/A	N/A	[33]
Ta/TaO_x_/Ru	–1.5 V	+2.0 V	>100	Abrupt	N/A	N/A	[50]
Pt/TaO_x_/n-Si	–2.5 V	+1.5 V	>10	Abrupt	N/A	N/A	[23]
Pt/TaO_x_/Ta	–0.8 V	+1.0 V	>10	Abrupt	N/A	N/A	[26]
Pt/TaO_x_/Ta	–0.8 V	+1.0 V	>10	Gradual	N/A	N/A	[18]
Ti/TaO_x_/HfO_2_/Pt	+1.5 V	–1.7 V	<10	Gradual	N/A	PD, STDP	[51]
TaN/Ta/TaO_x_/ Al_2_O_3_/Pt	+1.5 V	–1.8 V	<10	Gradual	N/A	PD	[52]
TiN/SiO_2_/ TaO_x_/Pt	+0.5 V	–1.5 V	>10	Gradual	N/A	PD, STDP	[31]
TaN/Ta/HfO_2_/ TaO_x_/Pt	+0.7 V	–0.9 V	>10	Gradual	N/A	PD	[1]
TiN/Ti/TaO_y_/HfO_x_/ Au	–5.0 V	+5.0 V	>10	Gradual	N/A	PD, STDP	[27]
W/Al_2_O_3_/TaO_x_/ TiN	+1.5 V	–2.2 V	<10	Gradual	N/A	N/A	[29]
Pt/TaO_x_/HfO_2_/Pt	+10 V	–5.0 V	>10	Gradual	N/A	N/A	[2]
Ta/WO_x_/TaOx/Pt	–4.0 V	+4.0 V	>10	Gradual	N/A	PD, PPF, SRDP	[17]
Pd/Ta_2_O_5_/TaO_x_/Pd	–1.8 V	+1.0 V	>10	Gradual	N/A	PD	[53]
ITO/WO_x_/TaO_x_/ TiN	+1.5	–1.8 V	>10	Gradual	Good (Figure 1d)	PD, STP, LTP, SADP, SRDP, PPF	This work

## Data Availability

Not applicable.
